# 11-Ketotestosterone and 11-Ketodihydrotestosterone in Castration Resistant Prostate Cancer: Potent Androgens Which Can No Longer Be Ignored

**DOI:** 10.1371/journal.pone.0159867

**Published:** 2016-07-21

**Authors:** Elzette Pretorius, Donita J. Africander, Maré Vlok, Meghan S. Perkins, Jonathan Quanson, Karl-Heinz Storbeck

**Affiliations:** 1 Department of Biochemistry, University of Stellenbosch, Stellenbosch, 7602, South Africa; 2 Proteomics Unit, Central Analytical Facility, University of Stellenbosch, Stellenbosch 7602, South Africa; Innsbruck Medical University, AUSTRIA

## Abstract

Dihydrotestosterone (DHT) is regarded as the most potent natural androgen and is implicated in the development and progression of castration resistant prostate cancer (CRPC). Under castrate conditions, DHT is produced from the metabolism of the adrenal androgen precursors, DHEA and androstenedione. Recent studies have shown that the adrenal steroid 11β-hydroxyandrostenedione (11OHA4) serves as the precursor to the androgens 11-ketotestosterone (11KT) and 11-ketodihydrotestosterone (11KDHT). In this study we comprehensively assess the androgenic activity of 11KT and 11KDHT. This is the first study, to our knowledge, to show that 11KT and 11KDHT, like T and DHT, are potent and efficacious agonists of the human androgen receptor (AR) and induced both the expression of representative AR-regulated genes as well as cellular proliferation in the androgen dependent prostate cancer cell lines, LNCaP and VCaP. Proteomic analysis revealed that 11KDHT regulated the expression of more AR-regulated proteins than DHT in VCaP cells, while *in vitro* conversion assays showed that 11KT and 11KDHT are metabolized at a significantly lower rate in both LNCaP and VCaP cells when compared to T and DHT, respectively. Our findings show that 11KT and 11KDHT are *bona fide* androgens capable of inducing androgen-dependant gene expression and cell growth, and that these steroids have the potential to remain active longer than T and DHT due to the decreased rate at which they are metabolised. Collectively, our data demonstrates that 11KT and 11KDHT likely play a vital, but overlooked, role in the development and progression of CRPC.

## Introduction

Prostate cancer (PCa) is the second most common cancer among men worldwide [1] with androgen deprivation therapy (ADT) being the first line treatment for advanced PCa since androgen signalling is essential for normal and malignant growth of prostate tissue. This treatment, which almost entirely eliminates circulating levels of testosterone (T), is initially effective. However, most men experience only short term regression (2–3 years), with nearly all patients developing the more aggressive castration-resistant PCa (CRPC) which is associated with poor survival rates [2].

The majority of evidence suggests that CRPC develops as a result of the reactivation of androgen receptor (AR) signalling despite castrate levels of T (≤50 ng/dL) [3–5]. The AR and AR-regulated genes are expressed in most clinical cases of CRPC demonstrating that the AR axis is reactivated and drives tumour growth [4,5]. Mechanisms proposed to be responsible for the continued AR activation include up-regulation of AR expression and/or gain-of-function mutations of the AR[6].

Recent clinical trials demonstrating beneficial clinical outcomes after treatment with the AR antagonist enzalutamide [7] and the CYP17A1 inhibitor abiraterone [8–10] have highlighted the continued androgen dependency of CRPC. Studies have confirmed that the adrenal androgen precursors, dehydroepiandrosterone (DHEA) and androstenedione (A4), serve as the source of intratumoral androgen production under castrate conditions [11–15]. The potent androgen, 5α-dihydrotestosterone (DHT), is produced by the alternate 5α-dione pathway, which bypasses T, to produce DHT via 5α-androstanedione (5α-dione) [12–15].

**Fig 1:**
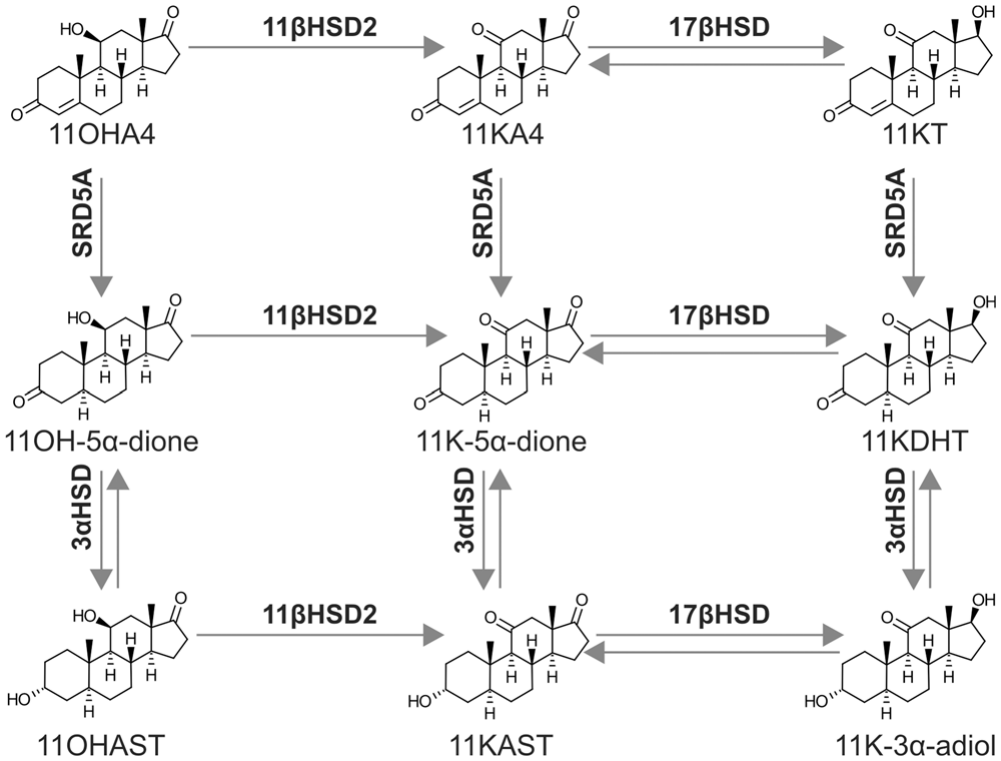
Biosynthesis of 11KT and 11KDHT from the adrenal androgen precursor 11OHA4. Enzymes: 11βHSD2, 11β-hydroxysteroid dehydrogenase; 17βHSD2, 17β-hydroxysteroid dehydrogenase; SRD5A1, steroid 5α-reductase type 1; 3αHSD2, 3α-hydroxysteroid dehydrogenase. Steroids: 11OHA4, 11β-hydroxyandrostenedione; 11KA4, 11-ketoandrostenedione; 11KT, 11-ketotestosterone; 11OH-5α-dione, 11OH-5α-androstanedione; 11K-5α-dione, 11-keto-5α-androstanedione; 11KDHT, 11-ketodihydrotestosterone; 11OHAST, 11β-hydroxyandrosterone; 11KAST, 11-ketoadrenosterone; 11K-3α-adiol, 11-keto-5α-androstane-3α,17β-diol.

In addition to DHEA and A4, the human adrenal gland produces substantial amounts of the inactive C19 steroid 11β-hydroxyandrostenedione (11OHA4) [16–18] via the cytochrome P450 11β-hydroxylase (CYP11B1) catalysed hydroxylation of A4 [19,20]. 11OHA4 is one of the most abundant C19 steroid produced by the human adrenal, both before and after adrenocorticotrophic hormone (ACTH) treatment [17]. A recent study by our laboratory identified a novel pathway for 11OHA4 metabolism in androgen dependent prostate cancer cells, which leads to the production of the androgens 11-ketotestosterone (11KT) and 11keto-5α-dihydrotestosterone (11KDHT) (Fig 1). We showed that at the concentration of 1 nM, 11KT and 11KDHT have androgenic properties comparable to T and DHT, respectively [21]. However, further work is needed to characterize these androgens.

The aim of this study was therefore to compare the androgenic properties of 11KT and 11KDHT to that of T and DHT. Competitive whole cell binding assays revealed that 11KT and 11KDHT bind to the human AR with affinities similar to that of T and DHT. Transactivation assays on a synthetic androgen response element (ARE) demonstrated that the relative agonist potencies and efficacies of 11KT and 11KDHT are comparable to that of T and DHT, respectively. Moreover, we showed that 11KT and 11KDHT treatment of two androgen dependent prostate cancer cell lines, LNCaP and VCaP, result in the regulation of endogenous AR-regulated genes at both the mRNA and protein level, and also drive cellular proliferation. Finally, we demonstrate that 11KT and 11KDHT are metabolised at a lower rate than T and DHT in both LNCaP and VCaP cells and as a result are likely able to exert prolonged androgenic effects. These findings confirm that both 11KT and 11KDHT are *bona fide* androgens and suggest that the 11OHA4 pathway may be a potential role player in the development and progression of CRPC.

## Materials and Methods

### Steroids

11KT, 11KDHT and mibolerone (Mib) were purchased from Steraloids. T and DHT were purchased from Sigma-Aldrich, while [H^3^]-Mib (76.8 Ci/mmol) was purchased from Perkin Elmer. All steroids were dissolved in absolute ethanol and added to the culturing medium at a final concentration of no more than 0.1% ethanol. Cortisol-9, 11, 12, 12-d4 (cortisol-d9), testosterone-1, 2-d2 (T-d2), progesterone-2, 2, 4, 6, 6, 17α, 21, 21, 21-d9 (P4-d9) and 4-pregnen-17α-ol-3,20-dione-2, 2, 4, 6, 6, 21, 21, 21-d8 (17OHP4-d8) were purchased from Cambridge Isotope Laboratories. Gestodene (GES) and drospirenone (DRSP) were purchased from Sigma-Aldrich.

### Plasmid constructs

The reporter construct for selective androgen response elements (AREs), 4xSC ARE1.2 [22] and the plasmid expressing the human androgen receptor, pSVARo [23] were obtained from Prof. F. Claessens (University of Leuven, Belgium).

### Whole cell binding assays

COS-1 cells were purchased from the American Type Culture Collection (ATCC) and were cultured in Dulbecco’s modified Eagle’s medium (DMEM) supplemented with 10% fetal calf serum (FCS) and 1% penicillin-streptomycin. Cultures were maintained in 75 cm^2^ culture flasks (Greiner Bio-One International) at 37°C, in an atmosphere of 90% humidity and 5% CO_2_. Competitive whole cell binding assays were performed in COS-1 cells as previously described by Africander *et al*. [24]. Using the dissociation constant (*K*_d_) for Mib reported in Africander *et al*. [24], the *K*_i_ values for DHT, 11KDHT, T and 11KT were determined from heterologous displacement curves using the equation by Swillens [25] which takes ligand depletion into account.

### Luciferase reporter assays

COS-1 cells were seeded into 10 cm^2^ dishes at a density of 2 x 10^6^ cells per dish. Following a 24 hour incubation, cells were co-transfected with 9 μg luciferase reporter construct (4*xSC* ARE1.2) and 0.9 μg hAR expression construct (pSVARo) using XtremeGene HP transfection reagent (Roche). Cells were incubated for 24 hours and subsequently replated into 24-well Corning^®^ CELLBIND^®^ surface plates (Corning, NY, USA) at a density of 1 X 10^5^ cells per well. The following day, cells were treated with increasing concentrations of DHT, T, 11KDHT or 11KT in serum-free DMEM. Cells were lysed and analysed as previously described [26], with the exception that the protein concentration in the lysate was determined by the Pierce BCA method (Pierce Chemical).

### Prostate cancer cell lines

LNCaP cells, purchased from the European Collection of Cell Cultures (ECACC), were cultured in RPMI-1640 media supplemented with 10% FCS and 1% penicillin-streptomycin and at all stages cultured using Corning^®^ CELLBIND^®^ surface plates. VCaP cells were purchased from the American Type Culture Collection (ATCC) and were cultured in DMEM supplemented with 10% FCS, 1% sodium pyruvate and 1% penicillin-streptomycin in 75 cm^2^ culture flasks (Greiner Bio-One International). LNCaP and VCaP cells were authenticated by the ECACC and ATCC, respectively, using the PCR of short tandem repeat sequences within chromosomal microsatellite DNA (STR-PCR). Both cell lines were cultured at 37°C, in an atmosphere of 90% humidity and 5% CO_2_ and were passaged for fewer than 6 months from the time of resuscitation.

### RNA isolation and qPCR

LNCaP and VCaP cell lines were plated at a density of 4 X 10^5^ cells per well into 12-well plates. The following day, cells were treated for 24 hours with 1 or 10 nM of the appropriate steroid in media supplemented with 10% CS-FCS. The AR inhibitor bicalutamide (1 μM) (Sigma-Aldrich) was included as a negative control. Total RNA was isolated using a Direct-zol^™^ RNA MiniPrep kit (Zymo Research) and cDNA subsequently synthesized using a Transcription First Strand cDNA synthesis kit (Roche). qPCR was performed using a LightCycler 96 instrument and the KAPA SYBR^®^ FAST qPCR Master Mix for LightCycler^®^ (KAPA Biosystems). Primer sequences were as follows, *KLK3* [27]: 5’-AGGCCTTCCCTGTACACCAA-3’ (forward) 5’-GTCTTGGCCTGGTCATTTCC-3’ (reverse), *FKBP5* [28]: 5’-GAATACACCAAAGCTGTTGA-3’ (forward) and 5’-CTCTTCCTTGGCATCCT-3’ (reverse), *TMPRSS2* [28]: 5’-CTGCCAAGGTGCTTCTC-3’ (forward) and 5’-TTAGCCGTCTGCCCTC-3’ (reverse). Reference genes: *PBGD* [28]: 5’-CATGTCTGGTAACGGCAATG-3’ (forward) and 5’GTACGAGGCTTTCAATGTTG-3’ (reverse), *ALAS* [29]: 5’-TTCCACAGGAGCCAGCATAC-3’ (forward) and 5’-GGACCTTGGCCTTAGCAGTT-3’ (reverse). PCR efficiency exceeded 90% for all primer sets.

### Protein quantification by LC-MS

#### Sample preparation

VCaP cells were plated at a density of 4 X 10^6^ cells per 10 cm dishes and incubated with media supplemented with 10% CS-FCS for 48 hours prior to treatment with 1 nM DHT, T, 11KDHT, 11KT or a vehicle control. After 48 hours, cells were collected, washed with PBS three times, weighed, and stored at -80°C until use.

Cells were thawed and proteins extracted using an extraction buffer containing; 100 mM NaCl, 2 mM EDTA, 6 M guanidine-HCl, 1% octylgluco-mano-pyranoside (OGP) and 5 mM triscarboxyethyl phosphine (TCEP) in 100 mM triethylammonium bicarbonate (TEAB) (pH = 8). After centrifugation, the remaining pellet was resuspended in a second extraction buffer containing; 1% taurocholic acid and 1 M NaCl in 100 mM TEAB (pH = 8). The supernatants from each extraction were pooled and an overnight acetone precipitation was performed. Thereafter, the samples underwent centrifugation and remaining supernatants were treated with 5% phosphotungstic acid. The pellets were air dried and dissolved in 100 mM TEAB containing 4 M guanidine-HCl and 1% OGP. Protein concentrations were determined spectrophotometrically. Samples were reduced using 50 mM TCEP in 100 mM TEAB followed by the modification of reduced cystein residues using methyl methanethiosulfonate (MMTS). Thereafter, the samples were diluted with 100 mM TEAB and proteins digested using trypsin. After being dried and resuspended in 2% acetonitrile containing 0.1% formic acid, the samples were desalted using C18 stage tips.

#### LC-MS

Liquid chromatography was performed using a Thermo Scientific Ultimate 3000 RSLC equipped with a C18 trap column and a C18 analytical column. Samples were analysed using a Thermo Scientific Orbitrap Fusion Tribrid mass spectrometer equipped with a Nanospray Flex ionization source. The raw files generated were imported into Proteome Discoverer v1.4 (Thermo Scientific) and processed using the Mascot and SequestHT algorithms. Peptide validation was performed using the percolator node set to search against a decoy database with a strict false discovery rate (FDR) of 1%. Additional analyses were performed using the X! Tandem Sledgehammer algorithm. Output files from the three algorithms were combined and analysed using Scaffold (Proteomesoftware).

### Proliferation assays

LNCaP cells were plated at 2 X 10^4^ cells per well in Corning^®^ CELLBIND^®^ 96-well surface plates, while VCaP cells were plated at 4 X 10^4^ cells per well in 96-well plates (Greiner Bio-One International). The cells were incubated for 72 h after which the media was replaced with media supplemented with 10% CS-FCS and the cells incubated for a further 24 h. Steroids were subsequently added to obtain final concentrations of 0.1, 1 and 10 nM. LNCaP cells were incubated for 7 days following the addition of steroid, while VCaP cells were incubated for 10 days. Cell growth was subsequently assessed using a resazurin assay [30,31].

### Steroid metabolite analysis

#### Sample preparation

LNCaP and VCaP cells were plated at 2 X 10^5^ cells per well in 12-well Corning^®^ CellBIND^®^ surface plates. After 24 h, the media was replaced with media supplemented with 10% CS-FCS and incubated for an additional 24 h. Cells were treated with 100 nM (DHT, 11KDHT) or 10 nM (T, 11KT) as well as a vehicle control and 1 ml aliquots taken at specific time intervals (6, 12, 24, 48 and 72 h). An internal standard mix containing 15 ng cortisol-d4, 1.5 ng T-d2, 15 ng 17OHP4-d9, 15 ng P4-d9, 12.4 ng GES and 14.7 ng DRSP were added to samples prior to extraction. Samples were subsequently extracted with 3 ml methyl tertiary butyl ether (MTBE). After vortexing for 10 min and centrifugation for 5 min, the aqueous layer was frozen at -80°C and the organic layer transferred to a clean test tube. Steroids were dried under a stream of nitrogen and resuspended in 50% methanol prior to analysis by UPC^2^- MS/MS.

#### UPC^2^-MS/MS

Steroids were analysed by ultra-performance convergence chromatography-tandem mass spectrometry (UPC^2^-MS/MS). Steroid metabolites were separated using an Acquity UPC^2^ system (Waters Corporation, Milford, USA) with an Acquity UPC^2^ BEH 2-EP column (3 mm X 100 mm, 1.7 μm particle size). The mobile phase consisted of liquid CO_2_ modified with methanol. Separation was achieved using a 4 minute linear gradient from 2% to 9.5% methanol at a constant flow rate of 2.0 mL.min^-1^. The column temperature and automated back pressure regulator (ABPR) were set to 60°C and 2000 psi, respectively. The steroids were quantified using a Waters Xevo triple quadrupole mass spectrometer (Waters, Milford, USA). Steroids were measured in multiple reaction monitoring (MRM) mode using electrospray in the positive ionization mode (ESI+). Calibration curves were constructed by using weighted (1/x2) linear least squares regression. Data was collected with MassLynx (version 4.1) software (Waters, Milford, USA).

### Statistical analysis

The Graph Pad Prism^®^ software (Version 6) was used for data manipulations, graphical representations and statistical analysis. Non-linear regression and one site competition were used in whole cell binding assays, while non-linear regression and sigmoidal dose response were used in transactivation experiments. The effect of steroid treatment on mRNA expression and cellular proliferation were analysed using a Student's t tests and one-way ANOVA followed by Dunnett's multiple comparisons test, respectively. All steroid treatments were compared to the vehicle control. Statistically significant differences are indicated by *, ** or *** for p<0.05, p<0.01 or p<0.001, respectively. The effect of steroid treatment on protein expression was analysed in Scaffold (Proteomesoftware) using a Student’s t test. Statistically significant differences are indicated by * for p<0.05.

## Results

### 11KT and 11KDHT bind to the AR with affinities similar to that of T and DHT

**Fig 2:**
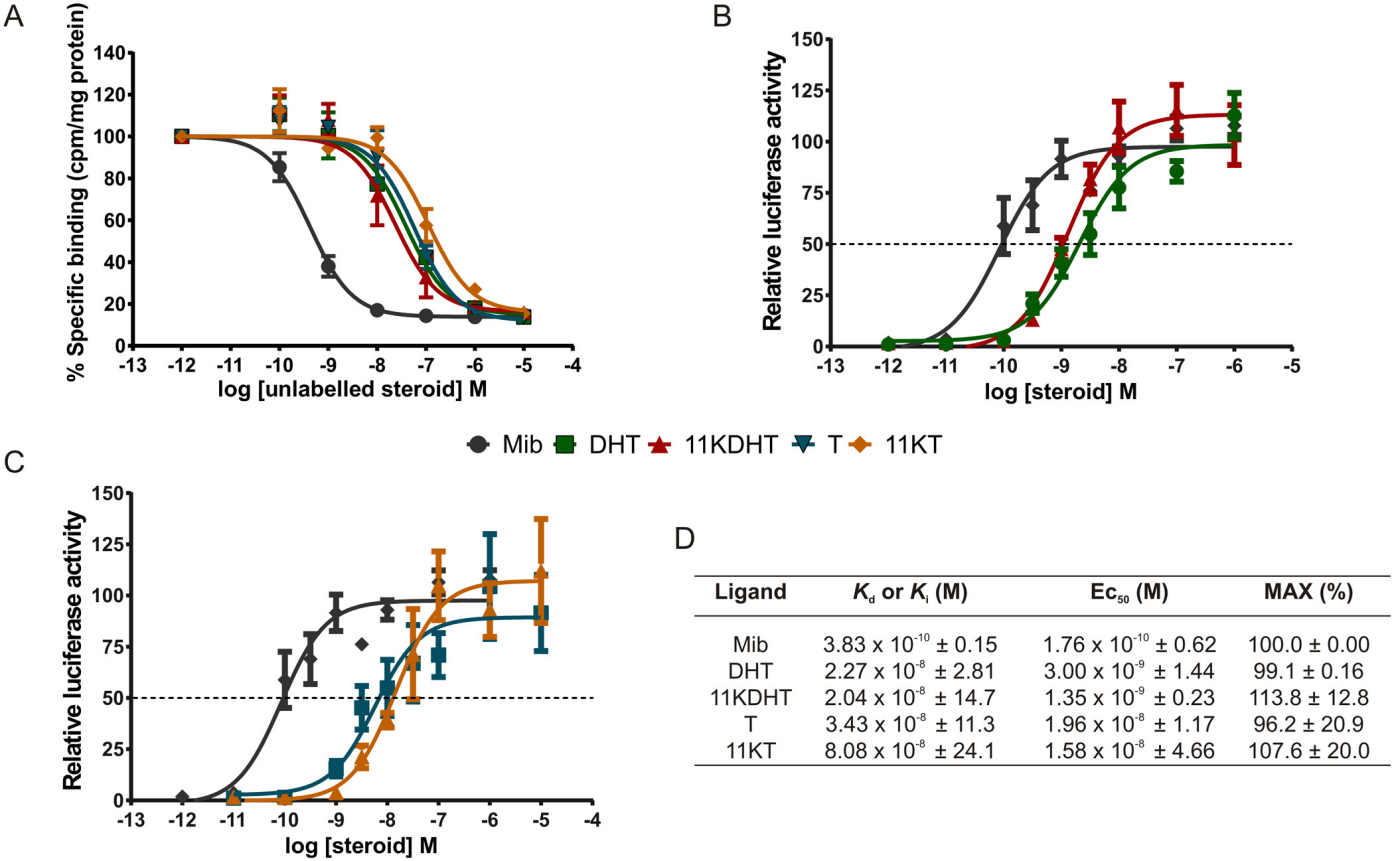
Binding of DHT, T, 11KDHT and 11KT to the human AR (A) and transactivation via an ARE (B and C). Binding affinities, agonist potencies and efficacies of DHT, 11KDHT, T and 11KT relative to the synthetic AR agonist mibolerone are summarised in (D). Whole cell binding assays (A) were conducted in COS-1 cells transiently transfected with pSVARo. Cells were incubated with 0.2 nM [^3^H]-Mib in the absence and presence of increasing concentrations of either unlabelled Mib, DHT, 11KDHT, T and 11KT for 16 hours. Results are plotted as % specific binding where the total specific binding of [^3^H]-Mib only is set to 100% and binding of unlabelled steroid is set as a % binding relative to that. Whole cell binding results are shown as means ± SEM of three independent experiments performed in triplicate. Transactivation assays (B and C) where performed in COS-1 cells transiently transfected with the pSVARo expression vector and the 4xSC ARE1.2-luc reporter. Agonist activity was measured by incubating cells in the presence of increasing concentrations of either Mib, DHT, T, 11KDHT or 11KT for 24 h. Induction is shown as % luciferase activity expressed in relative light units (rlu’s), with the maximal response of Mib (10^−5^ M) set to 100%. Luciferase assays are shown as means ± SEM of six independent experiments performed in quadruplicate.

The results indicate that 11KT (*K*_i_ = 80.8 nM) and 11KDHT (*K*_i_ = 20.4 nM) bind to the human AR with affinities similar to that of T (*K*_i_ = 34.3 nM) and DHT (*K*_i_ = 22.7 nM) (Fig 2A and 2D). The affinity of these ligands for the AR was approximately 100-fold lower than that of the synthetic androgen, Mib (*K*_d_ = 0.38 nM).

### 11KT and 11KDHT are full AR agonists

In light of the observation that 11KT and 11KDHT bind to the AR with affinities similar to that of T and DHT, and our previous finding that 11KT and 11KDHT act as AR agonists at a physiologically relevant concentration of 1 nM [21], we set out to determine the relative agonist potency and efficacy of 11KT and 11KDHT for transactivation. Using COS-1 cells transiently transfected with a human AR expression vector and a selective-AR androgen response element (ARE) driven luciferase reporter construct, we show that 11KT and 11KDHT display similar maximal induction (p > 0.05) as Mib and DHT, confirming that they are both full AR agonists (Fig 2). Of note, the potency determined for 11KDHT (1.3 nM) was statistically equal to that of DHT (3.0 nM) which is considered to be the most potent natural androgen (Fig 2C and 2D). Moreover, the efficacies of 11KDHT (113.84%) and DHT (99.14%) were not statistically different. Similarly potency and efficacy of 11KT (15.8 nM; 107.59) were not statistically different to that of T (19.6 nM; 96.21) (Fig 2B and 2D).

### 11KT and 11KDHT induce AR-regulated gene expression

#### mRNA expression

In order to investigate if 11KT and 11KDHT demonstrate androgenic activity on endogenous AR-regulated genes, the mRNA expression levels of *KLK3* (NM_001030047), *FKBP5* (NM_001145775) and *TMPRSS2* (NM_001135099) were assessed in two androgen dependent prostate cancer cell lines, LNCaP and VCaP. LNCaP cells express a mutated AR (T877A) [32], while VCaP cells express the wild type AR. A twenty-four hour time point was chosen as significant upregulation of *KLK3* and *TMPRSS2* has previously been demonstrated at this time point in both LNCaP and VCaP cells [33,34].

**Fig 3:**
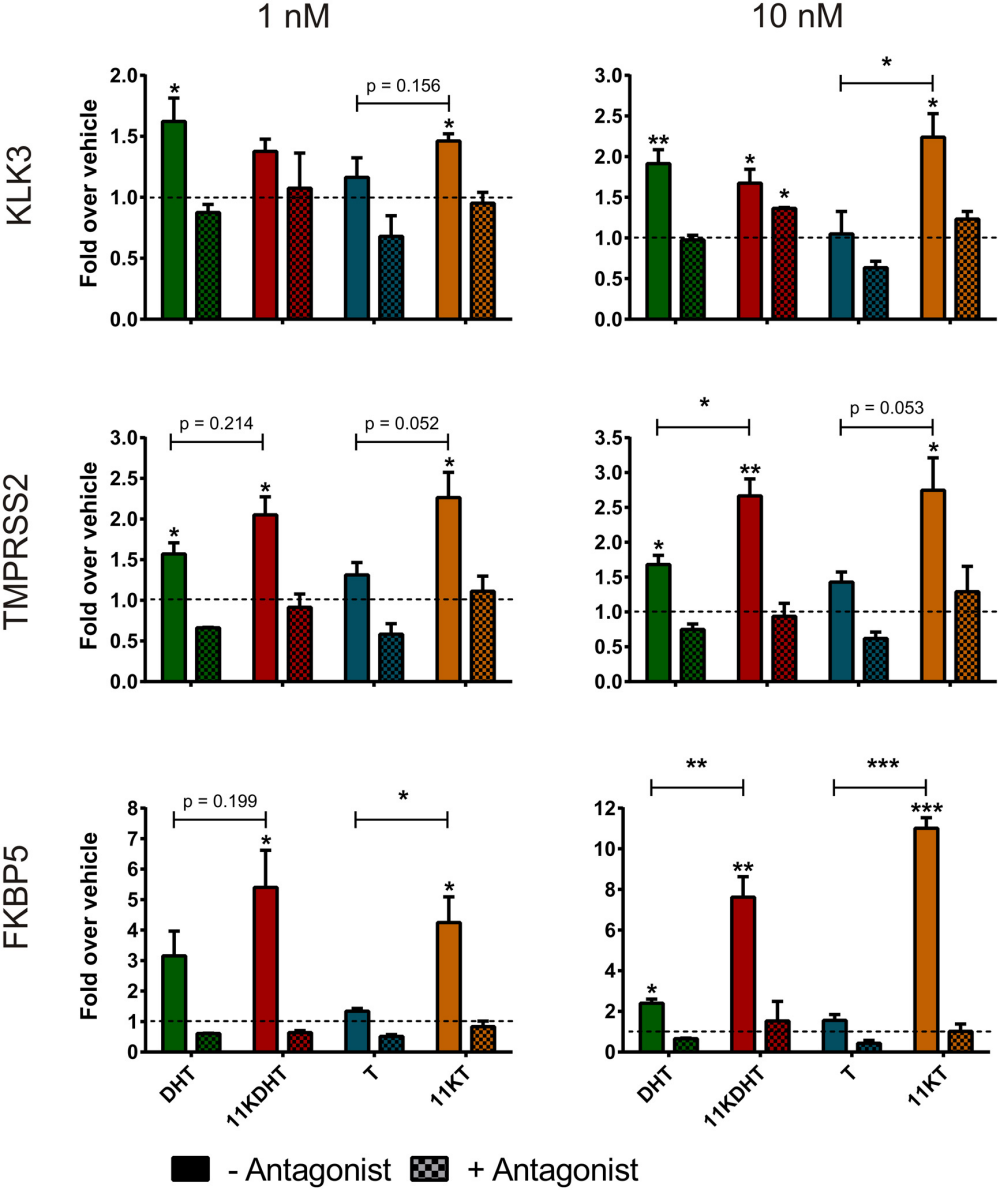
Induction of AR-regulated gene expression in LNCaP cells by DHT, 11KDHT and 11KT. Cells were incubated with CS-FCS-supplemented media for 24 hours prior to treatment with 1 or 10 nM steroid for an additional 24 hours prior to analysis by qPCR. Gene expression was calculated relative to the geometric mean of the reference genes *ALAS* and *PBGD*. Fold change over vehicle was calculated using the method described by Pfaffl et al [48]. Results are shown as means ± SEM of three independent experiments performed in triplicate. Data from individual experiments was all normalized using log transformation and mean-centering prior to analysis [49].

**Fig 4:**
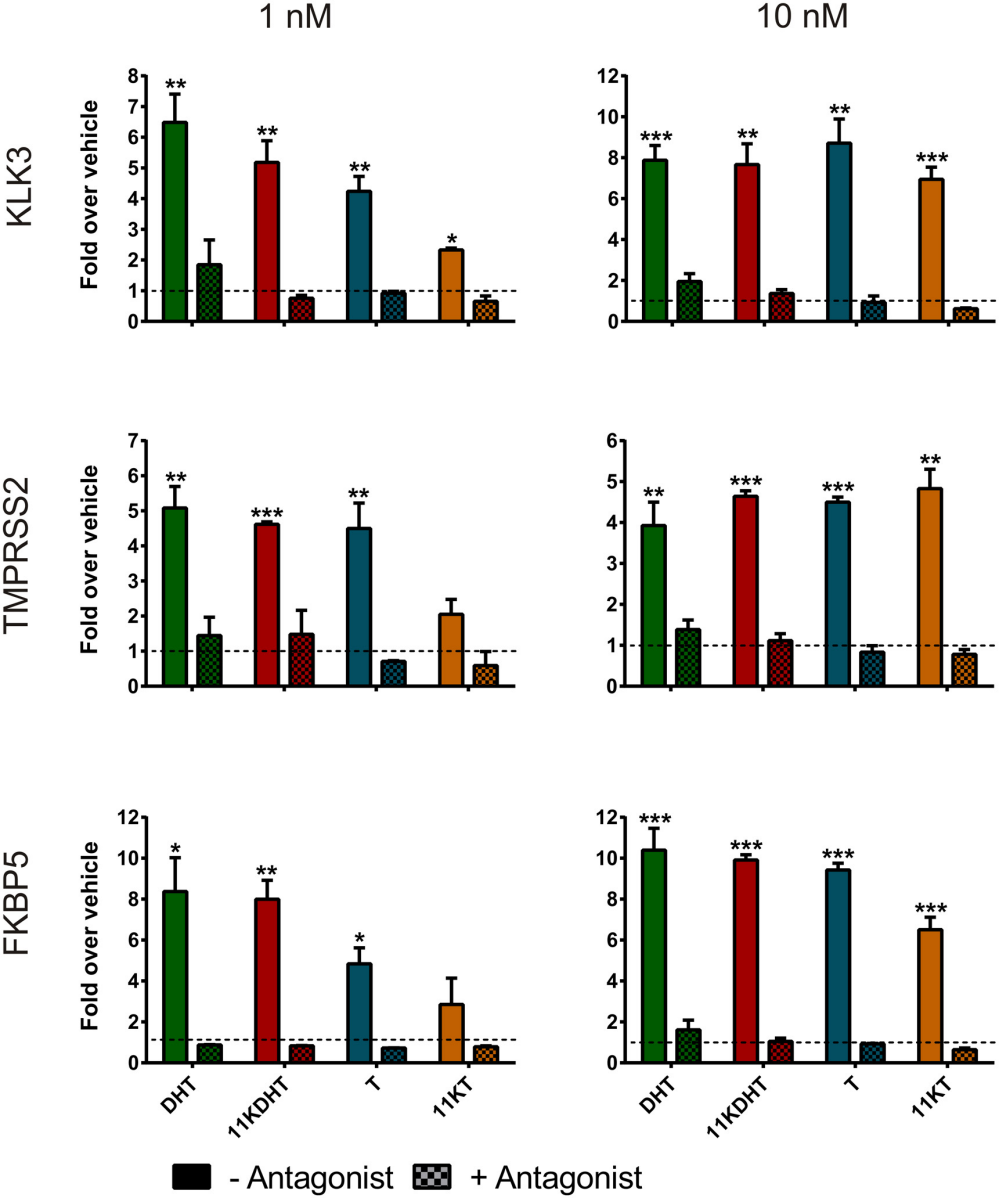
Induction of AR-regulated gene expression in VCaP cells by DHT, 11KDHT, T and 11KT. Cells were incubated with CS-FCS supplemented media for 24 hours prior to treatment with 1 or 10 nM steroid for an additional 24 hours prior to analysis by qPCR. Gene expression was calculated relative to the geometric mean of the reference genes *ALAS* and *PBGD* and are expressed as the fold change over the vehicle control. Results are shown as means ± SEM of three independent experiments performed in triplicate. Data from individual experiments was all normalized using log transformation and mean-centering prior to analysis [49].

Treatment with 1 or 10 nM 11KDHT resulted in the significant upregulation of *KLK3*, *TMPRSS2* and *FKBP5* in both LNCaP (Fig 3) and VCaP (Fig 4) cells, with the exception of *KLK3* at 1 nM in LNCaP cells (p = 0.0529) (Fig 3). Consistent with a previous study [28], we show that all three endogenous AR-regulated genes were significantly upregulated by DHT in both cell lines, with the exception of *FKBP5* at 1 nM in LNCaP cells (p = 0.0579) (Fig 3). Interestingly, treatment with T did not upregulate the mRNA expression of either *KLK3*, *TMPRSS2* or *FKBP5* in LNCaP cells at either test concentration, but significantly upregulated their expression at both test concentrations in VCaP cells (Fig 4). In contrast, 11KT treatment resulted in the upregulation of all of the AR-regulated genes in LNCaP cells at both 1 and 10 nM.

While 1 nM 11KT only significantly upregulated the expression of *KLK3* in VCaP cells, 10 nM 11KT upregulated all three AR-regulated genes significantly. This data confirms that both 11KDHT and 11KT are androgenic and are able to regulate the mRNA expression of endogenous AR-regulated genes. We confirmed that any observed changes in gene expression were due to AR activation by including a control for each test compound in the presence of the competitive AR inhibitor, bicalutamide. No significant changes in gene expression were observed upon steroid treatment in these samples with the exception of *KLK3* in LNCaP cells treated with 10 nM 11KDHT (Figs 3 and 4). Finally, it is worth noting that in LNCaP cells the induction of *KLK3*, *TMPRSS2* and *FKBP5* expression by 11KDHT and 11KT tended to result in a greater fold induction than that observed for DHT or T, respectively (Fig 3). The differences between 10 nM DHT and 11KDHT were statistically significant for both *TMPRSS2* and *FKBP5*. Interestingly, differences between T and 11KT induced expression were statistically significant for *KLK3* (10 nM treatment) and *FKBP5* (1 and 10 nM treatments), and approached significance for *TMPRSS2* (p = 0.052, 1 nM treatment; p = 0.053, 10 nM treatment).

#### Protein expression

The qPCR results described above showed that a robust response to androgen treatment was achieved in VCaP cells. Considering that VCaP cells are an accepted cell model for CRPC [35], while LNCaP cells are not [36], we selected VCaP cells for proteomic analysis. Cells were treated with 1 nM DHT, 11KDHT, T or 11KT, proteins were extracted and analysed using LC-MS. A total of 1439 proteins were identified which were common to all treatments. We subsequently determined the fold change in protein expression (relative to the vehicle control) for twenty-seven known AR-regulated proteins (Table 1). Of these, the expression of twenty-two selected proteins (ABCE1, ACACA, ACLY, ACSL3, BCAM, COPA, CPT1A, DCXR, FKBP5, GSK3A, IDI1, MCM7, NAMPT, PDIA3, PDIA4, PSA, PSMD2, PSMD3, RAB7A, RDH11, RPN1 and UGDH) are known to be upregulated by androgens. The number of these proteins which were significantly upregulated by steroid treatment were 3/22 for DHT, 13/22 for 11KDHT, 19/22 for T and 17/22 for 11KT. Notably, FKBP5 was significantly upregulated by DHT (2.5-fold), 11KDHT (4.1-fold) and T (3.4-fold), but not 11KT. This result correlates with the qPCR data reported above (Fig 4). The remaining five proteins (AIM1, HNRPL, LIMA1, NONO and TOP1) are known to be downregulated by androgens. Four out of five of these proteins were downregulated by DHT, 5/5 by 11KDHT, 4/5 by T and 3/5 by 11KT.

**Table 1:** Regulation of AR-regulated proteins by DHT, 11KDHT, T and 11KT in VCaP cells. Cells were incubated with CS-FCS supplemented media for 48 hours prior to treatment with 1 nM steroid. Proteins were subsequently identified using mass spectrometry. Fold changes were calculated relative to the vehicle control. Statistically significant changes are indicated (P<0.05). Results are representative of three independent experiments.

ID	Protein	Fold change				References
		DHT	11K DHT	T	11KT	
**Upregulated by androgens**						
ABCE1	ATP-binding cassette sub-family E member 1	2.5	3.1	4.8*	2.8*	[50]
ACACA	Acetyl-CoA carboxylase 1	1.6	2.2	2.8*	2.0*	[50]
ACLY	ATP-citrate synthase	2.5	3.7*	4.6*	4.4*	[50]
ACSL3	Long-chain-fatty-acid—CoA ligase 3	2.0	5.4*	4.0*	2.3*	[50]
BCAM	Basal cell adhesion molecule	1.1	2.3*	2.4*	1.6*	[50]
COPA	Coatomer subunit alpha	1.4	1.8	2.4*	1.9*	[51]
CPT1A	Carnitine O-palmitoyltransferase 1, liver isoform	2.0	4.2*	11*	7.5*	[52]
DCXR	L-xylulose reductase	1.3	1.4	2.1*	1.7*	[51]
FKBP5	Peptidyl-prolyl cis-trans isomerase	2.5*	4.1*	3.4*	1.7	[50]
GSK3A	Glycogen synthase kinase-3 alpha	10*	3.7	_	13*	[50]
IDI1	Isopentenyl-diphosphate Delta-isomerase 1	2.5	4.2	6.8*	6.6*	[50]
MCM7	DNA replication licensing factor MCM7	0.8	2*	2.0*	2.2*	[50]
NAMPT	Nicotinamide phosphoribosyltransferase	2.5	2.5*	2.7*	2.3*	[50]
PDIA3	Protein disulfide-isomerase A3	1.0	1.4*	1.0	1.0	[50]
PDIA4	Protein disulfide-isomerase A4	1.1	1.5*	1.2	1.0	[50]
PSA	Puromycin-sensitive aminopeptidase	1.4	2.5*	3.8*	3.1*	[53]
PSMD2	26S proteasome non-ATPase regulatory subunit 2	2*	1.8	1.9*	1.5*	[54]
PSMD3	26S proteasome non-ATPase regulatory subunit 3	1.1	1.3	1.8*	1.6*	[51]
RAB7A	Ras-related protein Rab-7a	1.1	1.9*	1.9*	1.2	[50]
RDH11	Retinol dehydrogenase 11	3.3	6.2*	5.5*	2.9*	[50]
RPN1	Dolichyl-diphosphooligosaccharide—protein glycosyltransferase subunit 1	1.3	2.5*	2.2*	1.6*	[50]
UGDH	UDP-glucose 6-dehydrogenase	1.4	1.3	1.6*	1.2	[53]
**Downregulated by androgens**						
AIM1	Absent in melanoma 1 protein	0.3*	0.4*	0.6	0.8	[50]
HNRPL	Heterogeneous nuclear ribonucleoprotein L	0.6*	0.6*	0.7*	_	[51]
LIMA1	LIM domain and actin-binding protein 1	0.5	0.4*	0.4*	0.5	[50]
NONO	Non-POU domain-containing octamer-binding protein	0.8*	_	0.8*	0.8*	[51]
TOP1	DNA topoisomerase 1	0.6*	_	0.6*	_	[51]

*p<0.05

### 11KT and 11KDHT induce androgen dependent cellular proliferation

**Fig 5:**
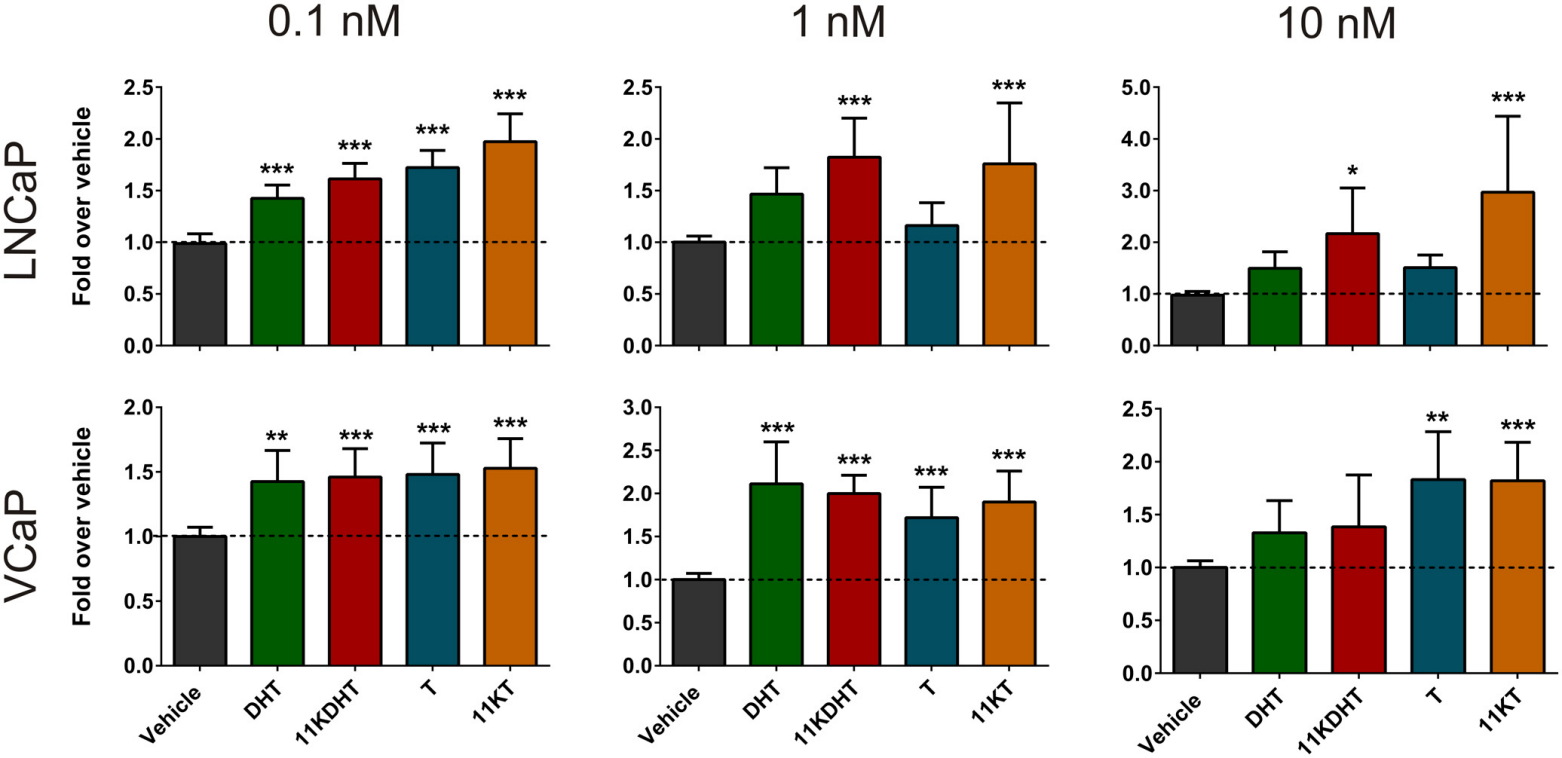
Induction of cell proliferation in LNCaP and VCaP cells by DHT, 11KDHT, T and 11KT. Cells were incubated with media supplemented with CS-FCS for 24 hours prior to treatment with 0.1, 1 or 10 nM steroids. Resazurin assays were carried out on day 7 (LNCaP) or day 10 (VCaP) after treatment. Results are shown as means ± SEM of three independent experiments with eight replicates each.

In order to assess the contribution of the novel steroids to the promotion of cell growth, the LNCaP and VCaP cell lines were treated with 0.1, 1 or 10 nM DHT, 11KDHT, T or 11KT (Fig 5). At 0.1 nM, all test steroids significantly induced LNCaP cell growth (DHT, 1.4-fold; 11KDHT, 1.6-fold; T, 1.7-fold and 11KT, 2.0-fold). At 1 and 10 nM, however, DHT and T no longer induced significant growth. This finding was not unexpected since androgen-induced cell growth is biphasic in LNCaP cells [37,38]. In contrast, both 11KDHT and 11KT induced significant cell proliferation, at both 1 nM (11KDHT, 1.8-fold; 11KT, 1.8-fold) and 10 nM (11KDHT, 2.2-fold; 11KT, 3.0-fold). In VCaP cells, all test steroids resulted in a significant increase in cellular proliferation at both 0.1 nM (DHT, 1.4-fold; 11KDHT, 1.5-fold; T, 1.5-fold; 11KT, 1.5-fold) and 1 nM (DHT, 2.1-fold; 11KDHT, 2.0-fold; T, 1.7-fold; 11KT, 1.9-fold). At 10 nM all steroid treatments appeared to stimulate cell growth, though this was only found to be significant for T and 11KT (T, 1.8-fold; 11KT, 1.8-fold).

### 11KT and 11KDHT are metabolised slower that T and DHT, respectively

In order to elucidate the potential mechanism by which 11KDHT and 11KT induce greater fold change in endogenous AR-regulated gene expression than DHT and T, respectively, we investigated the rate at which these steroids are metabolised by LNCaP and VCaP cells. Androgen inactivation is achieved by metabolism via 3α-hydroxysteroid dehydrogenases (3αHSD), producing inactive steroids, and/or glucuronidation catalysed by uridine 5'-diphospho-glucuronosyltransferase (UGT) enzymes [39]. LNCaP cells are known to express high levels of UGTs, while VCaP cells express lower levels of these enzymes [40,41]. UPC^2^-MS/MS was employed to measure the metabolism of 10 nM T and 11KT, and 100 nM DHT and 11KDHT. A 10-fold higher concentration of DHT and 11KDHT were chosen due to the poor ionisation of these steroids during analysis.

**Fig 6:**
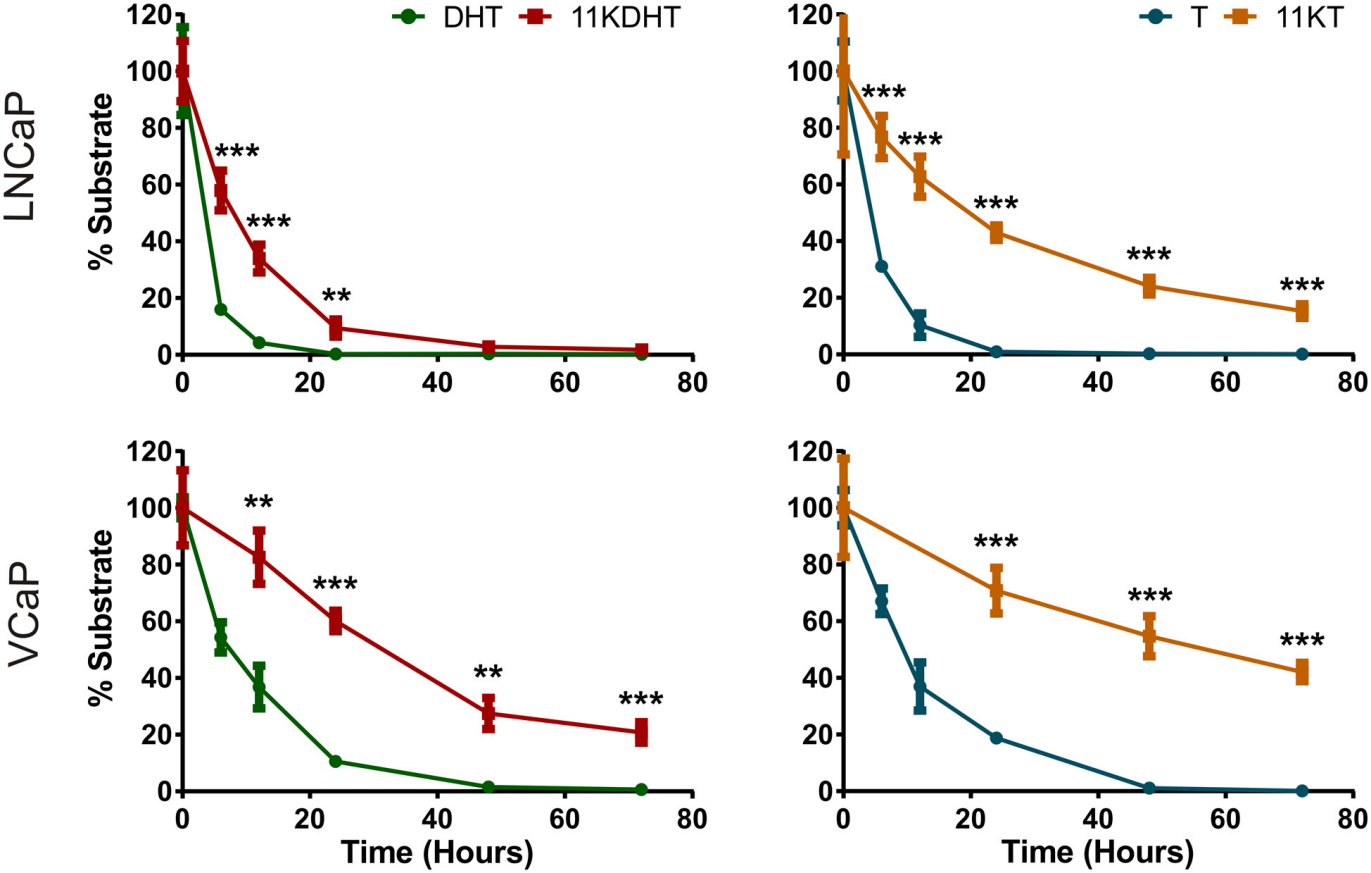
Metabolism of DHT, 11KDHT, T and 11KT by LNCaP and VCaP cells. Steroids were analysed by ultra-performance convergence chromatography-mass spectrometry (UPC^2^-MS/MS). Results are representative of two independent experiments performed in triplicate.

The results indicated a significant difference in the metabolism of 11KDHT and 11KT when compared to equivalent amounts of DHT and T, respectively (Fig 6). In LNCaP, 84% of the DHT substrate is metabolised in 6 hours, while only 42% of the 11KDHT is metabolised during the same period. Similarly 90% of the T is metabolised in 12 hours, while only 37% of the 11KT is metabolised.

A slower metabolic rate was observed in the VCaP cells. For example, DHT and T were depleted in 24 hours in LNCaP cells, while the complete metabolism of DHT and T by VCaP cells was only achieved after 48 hours. Nevertheless, the same trend was observed between the respective steroids. DHT was metabolised significantly faster (63% in 12 hours) than the same concentration of 11KDHT (17% in 12 hours). While DHT was fully metabolised after 48 hours, 21% of the 11KDHT remained detectable after 72 hours. The difference between T and 11KT metabolism in VCaP cells was also significant with only 30% of the 11KT being metabolised in 24 hours, while 81% of the T had been metabolised. T was depleted after 48 hours, while 42% of the 11KT remained after 72 hours.

## Discussion

CRPC is generally considered an androgen dependent condition [7–10,42]. There is overwhelming evidence indicating that the disease is able to survive castrate levels of T partly due to the availability of the adrenal androgen precursors DHEA and A4, which function as the substrates for intratumoral DHT production via the alternate 5α-dione pathway [12–15,43]. While the contribution of this pathway to the pool of active androgens is undoubtedly significant, the contribution of androgens from alternate sources, such as 11OHA4, cannot be ignored, especially considering that the human adrenal produces significantly more 11OHA4 than A4 [17]. The aim of this study was therefore to determine how the androgenic activity of the 11OHA4 metabolites, 11KT and 11KDHT, compare to that of the established androgens, T and DHT.

We first established the apparent *K*_i_ values of these steroids for the human AR, as well as their relative potencies and efficacies for transactivation via an ARE. Data showed that these steroids bind to the same site as that of Mib, and that 11KDHT binds to the AR with a similar affinity to that of T and DHT. While it appeared that the affinity of 11KT for the receptor was lower than that of 11KDHT, the difference was not statistically significant (Fig 2A and 2D). When comparing the potency and efficacy of these steroids using an AR-selective ARE, we demonstrated that both 11KT and 11KDHT are potent, efficacious AR agonists. The potency and efficacies of 11KT were comparable to that of T (Fig 2B and 2D) as has also previously been suggested [17,44]. To date, this is the first study to report the potency and efficacy of 11KDHT, and we furthermore showed that 11KDHT and DHT, the most potent natural androgen in mammals, are equipotent on the synthetic ARE-containing promoter used in this study (Fig 2C and 2D). These findings showing that DHT and T are not the only potent natural androgens, have significant implications for androgen dependent cancers such as CRPC. We therefore investigated whether the observed androgenic activity translated into the ability of these steroids to regulate endogenous AR-regulated gene expression and cell growth in the androgen dependent prostate cancer cell lines LNCaP and VCaP.

The results showing that 11KDHT and 11KT upregulated the expression of the endogenous AR-regulated genes, *KLK3*, *TMPRSS2* and *FKBP5* (Figs 3 and 4), and induced cell growth (Fig 5) in both the androgen-dependent LNCaP and VCaP cells confirm the status of 11KT and 11KDHT as *bone fide* androgens. The inclusion of the AR antagonist, bicalutamide, confirmed that the upregulation of *KLK3*, *TMPRSS2* and *FKBP5* gene expression by these steroids was AR dependent in both cell lines. Interestingly, the induction of *KLK3*, *TMPRSS2* and *FKBP5* gene expression by 11KT and 11KDHT in LNCaP cells was greater than that observed for T and DHT, respectively (Fig 3), highlighting the previously undetermined ability of these steroids to drive endogenous AR-regulated gene expression in mammalian cell lines. Similarly 11KT and 11KDHT induced significant cell growth in LNCaP cells at concentrations of 1 and 10 nM, while T and DHT failed to induce growth at the same concentrations (Fig 5). Although the induction of LNCaP cell growth by DHT and the synthetic androgen R1881 has previously been shown to be biphasic [37,38], a similar trend was not observed for 11KT and 11KDHTand thus requires further investigation in future.

We hypothesised that differences in the expression of endogenous AR-regulated genes, as observed in LNCaP cells (Fig 3), may in part be due to differences in the rates at which these androgens are metabolised. Active androgens, such as DHT and T, are inactivated by glucuronosyltransferase (UGT) catalysed glucuronidation, thereby blunting androgen signalling [45]. In addition, 5α-reduced steroids, such as DHT, can be inactivated by the action of 3α-hydroxysteroid dehydrogenases (3αHSD) prior to glucuronidation [18]. While T and DHT are metabolised by UGTs and 3αHSDs the efficiency with which 11KT and 11KDHT can be metabolised was uncertain. We therefore measured the decrease in 11KT and 11KDHT concentrations over time in both LNCaP and VCaP cells, and show that T and DHT were metabolised by both cell lines at a significantly higher rate than 11KT and 11KDHT, respectively (Fig 6). In addition, the rate of metabolism was significantly higher in LNCaP cells for all the steroids. This result correlates with the higher levels of UGT1A, UGT2B15 and UGT2B17 expressed in LNCaP cells as compared to VCaP cells [40,41]. The reduced rate of metabolism observed for 11KT and 11KDHT may allow these steroids to activate the androgen axis for a longer period than T and DHT, resulting in the increased expression of endogenous AR-regulated genes observed in the LNCaP cells (Fig 3).

The same trend was not, however, observed in VCaP cells when considering AR-regulated gene expression. Overall the upregulation of AR-regulated gene expression was greater in VCaP cells than LNCaP cells, which is not unexpected given both the lower rate of metabolism (Fig 6), due to reduced UGT expression [40,41], and the amplification of the AR gene in VCaP cells [46]. Proteomic analysis of AR-regulated protein expression did, however, reveal significant differences between DHT and 11KDHT treated cells. DHT exposure resulted in the significant regulation of only 7 out of the 27 AR-regulated proteins included in this study, while 11KDHT significantly regulated 18 of these proteins (Table 1). Since DHT and 11KDHT are equipotent and equally efficacious on a synthetic promoter (Fig 2C and 2D), the data suggests that the difference in protein expression may at least in part be due to differences in the rate of metabolism during the 48 hour induction period employed prior to proteomic analysis. T and 11KT resulted in the regulation of 23 and 20, proteins, respectively (Table 1). T and 11KT were also the only steroids to induce significant cell growth in VCaP cells at a concentration of 10 nM (Fig 5). While T and 11KT can be inactivated by glucuronidation, these steroids also serve as substrates for steroid 5α-reductase (SRD5A1), resulting in the production of the potent androgens DHT and 11KDHT. Like T and 11KT, the metabolic fate of DHT and 11KDHT is two-fold, however, in the case of DHT and 11KDHT both routes result in inactivation. These steroids are either glucuronidated directly or converted to the inactive metabolites 5α-androstane-3α,17β-diol (3α-adiol) and 11-keto-5α-androstane-3α,17β-diol (11K-3α-adiol) by 3αHSDs prior to glucuronidation [18].

In addition to the likely role played by 11KT and 11KDHT in CRPC, 11KT has recently been implicated in the androgen excess associated with classic 21-hydroxylase deficiency (21OHD). The levels of 11KT and other 11-oxygenated steroids were shown to be elevated significantly (3–4 fold) in patients with classic 21OHD when compared to healthy age matched controls. This finding, together with the observation that the routinely measured androgens, A4 and T (in woman), do not correlate well with the clinical evidence of androgen excess in 21OHD patients, led Turcu et al. [47] to propose that 11KT may be responsible for the androgen mediated effects associated with this condition.

## Conclusion

This study provides comprehensive evidence that 11KT and 11KDHT are potent and efficacious AR agonists, capable of driving gene regulation, protein expression and cell growth in androgen-dependent prostate cancer cells. The most novel and significant finding is that DHT and 11KDHT are equipotent and are equally efficacious, which highlights the fact that DHT may not be the only potent natural androgen. Differences in the rate at which these androgens are metabolised, with 11KT and 11KDHT being metabolised at a significantly lower rate than T and DHT respectively, have significant implications for androgen-dependent conditions such as CRPC. These findings highlight that not only can 11KT and 11KDHT activate the androgen axis, and in so doing drive cell growth, these steroids have the potential to remain active longer than T and DHT. Taking only intratumoral levels of DHT and T into account, as is currently the case in therapeutic approaches, could therefore lead to a substantial underestimation of AR activation in CRPC. Future studies should therefore focus on determining the physiological levels of 11KT and 11KDHT and assessing their contribution to CRPC as well as conditions resulting in androgen excess such as 21OHD.
